# Promotions for Medical Officers

**Published:** 1918-09

**Authors:** 


### PROMOTIONS FOR MEDICAL OFFICERS

The Chief-Surgeon has been working for some time to secure promotions for a large number of medical officers of the American Expeditionary Forces; but many obstacles have prevented ths consummation of his plans. He has, however, recently worked out a system, that has been approved by the Commander-in-Chief, which will give to many medical officers a deserved increase in rank; and which is based upon an equitable consideration of age, seniority, experience, special qualification and gallant or distinguished service.

Blanks have been sent to the Commanding Officers of Divisions, Hospitals and other Units for reports on the character of the services and qualifications of the Medical Reserve Officers. Upon receipt of these reports, and when the proportion of officers allowed the Medical Department of the American Expeditionary Forces by recent legislation is determined, the promotions will be made to those who deserve them in so far as it is possible to determine the merits of each individual case.

The General Staff of the Army will not allow promotions above first lieutenants to men of the draft age in any branch of the Army except for special qualifications or gallant service. Therefore few medical officers under thirty may hope for promotion at this time. They should not be discontented with this ruling however, because many lawyers and other professional men of the draft age who have had the best college training are serving in the ranks, while all worthy physicians of the draft age are officers. Since a medical officer's age is computed by his actual age plus one for each four months of service, the man who is twenty-nine and who has been in France for more than a year may hope for promotion. All lieutenants whose actual age is about thirty-one and who have completed one year's service will be eligible to promotion as captain. There are also a number of majors to be appointed. Not all officers above the age of thirty-one will be promoted to captain, nor will captains be given majorities unless the reports from their superior officers should show that their promotions are deserved.

The Chief Surgeon, as stated in a Circular, recognizes the inequalities in rank due to the fact that the Surgeon General's Office in Washington could not investigate fully the claims of each of the thousands of physicians who came into the service in a few months; and he is sincerely and earnestly striving to give to each medical officer in France the rank that he merits — in so far as the law allows him to make promotions in the different grades.

Medical officers of the American Expeditionary Force have been too busy in caring for the sick and wounded soldiers of our Army and in attending to their other daily duties to give much consideration to the question of rank. They have worked faithfully and without complaining in whatever capacity they have been called upon to serve, even though some have thought that their talents and attainments have not received the proper recognition in the matter of rank; but they will appreciate this effort of the Chief Surgon to give them the promotions which they deserve.

The extracts from the Circulars sent out by the Chief Surgeon bearing on the question of rank are published on page 267 ff. From them medical officers may decide whether or not they are entitled to promotion, and each may be assured that the Chief-Surgeon will carry out without partiality the plan which seems just and fair, and with due consideration for the facts in every case that is brought to his attention.

